# Comparative Characterization of MicroRNAs from the Liver Flukes *Fasciola gigantica* and *F. hepatica*

**DOI:** 10.1371/journal.pone.0053387

**Published:** 2012-12-31

**Authors:** Min-Jun Xu, Lin Ai, Jing-Hua Fu, Alasdair J. Nisbet, Qing-You Liu, Mu-Xin Chen, Dong-Hui Zhou, Xing-Quan Zhu

**Affiliations:** 1 State Key Laboratory of Veterinary Etiological Biology, Key Laboratory of Veterinary Parasitology of Gansu Province, Lanzhou Veterinary Research Institute, Chinese Academy of Agricultural Sciences, Lanzhou, Gansu Province, China; 2 National Institute of Parasitic Diseases, Chinese Center for Disease Control and Prevention, Shanghai, China; 3 College of Animal Science, South China Agricultural University, Guangzhou, Guangdong Province, China; 4 Parasitology Division, Moredun Research Institute, Midlothian, Scotland, UK; 5 Animal Reproduction Institute, Guangxi Key Laboratory of Subtropical Bioresource Conservation and Utilization, Guangxi University, Nanning, Guangxi Zhuang Nationality Autonomous Region, China; 6 College of Animal Science and Veterinary Medicine, Heilongjiang Bayi Agricultural University, Daqing, Heilongjiang Province, China; Wadsworth Center, United States of America

## Abstract

MicroRNAs (miRNAs) are key regulators of gene expression at the post-transcription level. The present study specifically explored and compared the miRNA expression profiles of *F. gigantica* and *F. hepatica* using an integrated sequencing and bioinformatics platform and quantitative real-time PCR. Nineteen and 16 miRNA candidates were identified from *F. gigantica* and *F. hepatica*, respectively. The two parasites shared 11 miRNAs, with 8 also showing similarity to miRNAs of *Schistosoma japonicum*. Another 8 miRNAs were identified as *F. gigantica*-specific and 5 as *F*. *hepatica-*specific, most of which were novel. Predicted target analysis with 11465 mRNA and EST sequences of *F. hepatica* and *F. gigantica* revealed that all of the miRNAs had more than one target, ranging from 2 to 398 with an average of 51 targets. Some functions of the predicted targets were only found in *F. gigantica*, such as “transcription regulator”, while some others were only found in *F*. *hepatica*, such as “reproduction” and “response to stimulus”, indicating the different metabolism and gene regulation patterns of the two parasites. The present study represents the first global comparative characterization of miRNA expression profiles of *F. gigantica* and *F. hepatica*, which has provided novel valuable resources for a better understanding of the two zoonotic trematodes.

## Introduction

The parasitic flatworms *Fasciola hepatica* and *F. gigantica* are common liver flukes which cause fasciolosis, a vector-borne disease with wide latitudinal, longitudinal and altitudinal dispersal in the world [1]–[3]. Fasciolosis can affect human beings and all ruminant animals of agricultural importance [4], [5]. The geographically wide distribution of these parasites makes fasciolosis one of the most important helminth diseases with a global distribution [6]–[9]. In addition to livestock health significance, fasciolosis is also an important food-borne zoonosis in humans. Conservative estimates suggest that 2.4–17 million people are infected with *Fasciola* spp. worldwide with a further 180 million at risk of infection. Most of these infected or “at-risk” populations are located in the Americas, parts of Europe, South Africa, the Middle East and Asia [10]–[12]. Having considerable human and animal health significance, flukes of the genus *Fasciola* are among the largest and best known digenean trematodes, with *F. gigantica* and *F. hepatica* as the two key representatives capable of causing significant public health problems in humans [13]. The transcriptome of *F. hepatica* was recently investigated and reported, providing an improved insight into biological processes and microRNA (miRNA) targets in *F.* *hepatica* [14].

miRNAs are 18–25 nucleotide non-coding RNAs that regulate gene expression at the post-transcriptional level. They are now considered as a key mechanism of gene regulation in organ development, cellular differentiation, proliferation, function and even regulation of the immune system [15]–[17]. miRNAs are essential for the complex life cycles of pathogenic parasites, allowing them to respond to environmental and developmental signals [18], [19]. The investigation of miRNA function in such pathogens can provide foundations for their control [20]. Due to the significance of miRNAs in parasite development and adaptation, and different environmental niches, there are likely to be differences in miRNA sequences and expression patterns between *F. hepatica* and *F. gigantica*, which will be of benefit in designing new tools for control. However, despite their socio-economic importance and the zoonotic significance of the two species, miRNA research on *F. hepatica* and *F. gigantica* has not previously been reported. Therefore, we compared the miRNA expression profiles of *F. hepatica* and *F. gigantica* here, by next-generation sequencing, bioinformatic analysis and stem-loop real-time reverse transcription polymerase chain reaction (qRT-PCR).

## Materials and Methods

### Ethics Statement

The buffalo and beef cattle from which *F*. *gigantica* and *F. hepatica* adults were collected respectively, were taken from two local abattoirs (Nanning Slaughterhouse, Nanning City, Guangxi Zhuang Autonomous Region, China; Wensu Slaughterhouse, Wensu County, Xinjiang Uygur Autonomous Region, China, respectively). These animals were being processed as part of the normal work of the two abattoirs.

### Parasites

Adults of *F*. *gigantica* and *F*. *hepatica* were collected from the gallbladders of slaughtered animals with naturally acquired infections. These worms were cleaned and stored as described previously [19]. Briefly, the worms were transferred to sterile physiological saline (37°C) in a sterile beaker, washed extensively with saline on a rotary shaker, transferred to Dulbecco’s modification of Eagle’s medium (DMEM) and incubated at 37°C (10% CO_2_) for 3 h to allow the flukes to regurgitate all the gut contents from their digestive tracts. Subsequently, the flukes were transferred to RNase-free screw-top cryotubes containing RNAlater (Sigma), kept at 4°C overnight and then stored at −80°C. The specific identity of each worm was verified as *F. hepatica* or *F. gigantica* by morphological features and by isolating genomic DNA [21] and conducting PCR-coupled, bidirectional sequencing (Shenggong Co. Ltd. Shanghai, China) of the internal transcribed spacers (ITS-1 and ITS-2) of nuclear ribosomal DNA [22].

### Small RNA Preparation and High-throughput Sequencing

Total RNA from three flukes of each species was prepared using Trizol reagent (Invitrogen) according to the manufacturer’s protocol with some modifications. For the flukes that were preserved in RNAlater solution, 50% isopropanol were used at the precipitation step to gain a clear mix solution. Ten µg total RNA were separated by electrophoresis on a Novex 15% TBE-Urea gel (Invitrogen Co. Ltd) and RNA in the region of 18–30 nt was purified. A reverse transcription PCR (RT-PCR) kit (Invitrogen Co. Ltd) was used for reverse transcription of the small RNAs to form complementary DNA (cDNA). Finally, this RT-PCR product was purified using a 6% TBE PAGE gel (Invitrogen Co. Ltd) and sequenced with a Solexa Genome Analyzer (Illumina HiSeq 2000) at Huada Genomics Institute Co. Ltd, China.

### High-throughput Sequencing and Computational Analysis

Clean reads were analyzed as described previously [19]. Briefly, adaptors, reads smaller than 18 nt and low quality sequences were trimmed from the raw data, and the reads were then searched against GenBank Rfam database (version 10.1) to remove non-coding RNAs including rRNA, tRNA, snRNA, snoRNA and other ncRNAs. RepeatMasker (http://www.repeatmasker.org) was used to eliminate repetitive sequences in the clean reads. The genome of *Schistosoma japonicum* (http://lifecenter.sgst.cn/schistosoma/cn) was used as the reference genome for short read alignment using the program SOAP [23]. miRNA candidates were identified with precursors which had standard fold-back structure. A stem-loop hairpin was considered typical when the mature miRNAs presented in one arm instead of the loop of hairpin precursors. The free energy hybridization threshold of a stem-loop hairpin was set lower than −18 kcal/mol. Conserved miRNAs homologues were identified by matching the miRNA candidates with miRNAs deposited in the miRBase database (version 16.0). miRNAs with no match were identified as “novel” miRNAs.

A total of 3055 and 8410 mRNA and EST sequences of *F. hepatica* and *F. gigantica* respectively were downloaded from NCBI, and were combined into a single dataset. Potential targets of miRNAs of the two parasites were predicted with RNAhybrid software [24]. To reduce false-positive results, two extra parameters were imposed on the analysis: 1) the △△G was set as lower than −25 kcal/mol; 2) the P-value was set as ≤0.01. The Gene Ontology database (GO, http://www.geneontology.org/) was used for functional analysis of predicted targets. In addition, published studies focusing on miRNA function were also searched to inform molecular function in the present work.

### Analysis of Novel miRNA Expression

The expression levels of novel miRNAs in adult *F. hepatica* and *F. gigantica* were analyzed with the modified stem-loop real-time quantitative reverse transcription polymerase chain reaction (qRT-PCR) using SYBR Green as described previously [25]. All of the primers were synthesized by Shenggong Co, Ltd., China. The PCR was performed using an ABI PRISM® 7300 Sequence Detection System and SYBR Green PCR Master Mix (TOYOBO). A 20 µl reaction mixture included 5 µl cDNA for each species (at 1∶20 dilution), 5 µM forward and reverse primers, and 10 µl 2× SYBR Green PCR Master Mix. The cel-miR-lin4 was added into the mixture as the endogenous control in all real time PCR reactions. The primer pairs were as follows: forward 5′-ACACTCCAGCTGGGTCCCTGAGACCTCAAGTG-3′ and reverse 5′-CTCAACTGGTGTCGTGGAGTCGGCAATTCAGTTGAGTCACACTT-3′. The cycling conditions were as follows: 95°C 5 min, followed by 30 cycles of 95°C for 15 s, 65°C for 15 s, and 72°C for 32 s. The quantification of each miRNA relative to the endogenous control was calculated using the equation: N = 2^–ΔCt^, ΔCt = Ct_miRNA_–Ct_lin4_ [26]. All reactions were carried out in triplicate.

## Results

### Profile Characteristics of Short RNAs from *F*. *hepatica* and *F*. *gigantica*

Deep sequencing yielded 16.41 and 10.36 million high-quality reads longer than 18 nt for *F. hepatica* and *F. gigantica*, respectively. Exons and introns accounted for a very small percentage of the clean reads, which indicated high integrity of the RNA in the samples. Among the reads, 14.4 (54.01%) million were common between the two species, and 8.7 and 3.5 million reads were *F.* *hepatica-* and *F. gigantica-*specific, which represented 2.9 and 0.48 million unique reads.

Non-coding RNAs (ncRNA), including rRNA, tRNA, snRNA, snoRNA, repeats, exons and introns, accounted for 4.3 (26.06%) and 2.5 (25.09%) million of the high-quality reads from *F. hepatica* and *F. gigantica*, respectively. The percentages of different kinds of ncRNA were similar for the two species. Repeat-associated small RNAs focused on two types of repeat: LINE/RTE:0 and LINE/RTE:1 in both species.

### Analysis of miRNAs Profiles

A total of 19 and 16 miRNA candidates with standard stem-loop structures were identified from *F*. *gigantica* and *F*. *hepatica*, respectively. Among them, 11 miRNAs were shared by the two parasites, including 8 conserved and 3 novel miRNAs. The conserved miRNAs all matched perfectly with those from *S*. *japonicum* in the miRBase database (Table 1). Furthermore, the 8 conserved miRNAs were identified as originating from 6 miRNA families, namely miR-124, miR-2 (miR-2b, miR-2e), bantam, miR-10, let-7 and miR-71 (miR-71, miR-71b).

**Table 1:** Comparison miRNA profiles of *Fasciola gigantica* and *F*. *hepatica.*

	Name	Mfe(kcal/mol)	Located at the 5p terminal	Located at the 3p terminal
**Shared miRNA**				
**Conserved** a				
1	sja-miR-12	−29.8	–	TAAGGCACGCGGTGAATGTCA
2	sja-bantam	−29.5	–	TGAGATCGCGATTAAAGCTGGT
3	sja-miR-2b	−33.6	–	TATCACAGCCCTGCTTGGGACAC
4	sja-miR-2e	−24.7	TACCAACTTAGACTGAGTTAT	TATCACAGTCCAAGCTTTGGT
5	sja-miR-10	−26.7	AACCCTGTAGACCCGAGTTTG	–
6	sja-let-7	−33.1	GGAGGTAGTTCGTTGTGTGG	–
7	sja-miR-71	−33.01	TGAAAGACGATGGTAGTGAGAT	–
8	sja-miR-71b	−36.2	TGAAAGACTTGAGTAGTGAGACG	–
**Novel** b				
9	miR-novel-share-03^c^	−30.1	TGGAAGCACTGTACAGCTGTTTT	–
10	miR-novel-share-02	−18.5	–	ATGAAACAGCTGTACAGTGC
11	miR-novel-share-04	−22.2	–	GCCTCCATAGCTCAGTGGTCAGA
**Species-specific miRNA**				
***F. gigantica*** **-specific**				
1	Fgi-cin-miR-4006b	−27.5		TGGAACAATGTAGGTAAGGG
2	Fgi-miRNA-novel-01	−19	–	ATGGATGGATAGATGGATGG
3	Fgi-miRNA-novel-06	−21.3	–	GAGGTAGGTGAAGTGGTCGA
4	Fgi-miRNA-novel-10	−24.4	–	TCCATCATCATCATCATCATCATC
5	Fgi-miRNA-novel-09	−27.5	–	TGGAACAATGTAGGTAAGGG
6	Fgi-miRNA-novel-05	−18	GACCACTTCACCTACCTCGG	–
7	Fgi-miRNA-novel-03	−33.5	GACGGGGTGGCCGAGTGGTTA	–
8	Fgi-miRNA-novel-15	−23.7	GAGAGAGAGAGAGAGGGAGAGA	–
***F. hepatica*** **-specific**				
1	Fhe-mmu-miR-1957	−23.5	–	CAGTCGGTAGAGCATCAGACT
2	Fhe-miRNA-novel-01	−20.2	–	AAACAGCTGTACAGTGCTTCT
3	Fhe-miRNA-novel-08	−18.4	–	TGATGATGGATTTACTGTTGT
4	Fhe-miRNA-novel-07	−21.6	ACGATGATGATGATGATGATTT	–
5	Fhe-miRNA-novel-10	−19.24	GATGGAGTAGCTATGGGGTCT	–

Note:

a miRNA candidates of *F*. *gigantica* and *F*. *hepatica* matched with that of other species deposited in the miRBase;

b miRNA candidates of *F*. *gigantica* and *F*. *hepatica* matched with no miRNAs deposited in the miRBase;

c novel miRNA candidates shared by *F*. *gigantica* and *F*. *hepatica*; Fgi: *F*. *gigantica*; Fhe: *F*. *hepatica*.

A total of 8 and 5 miRNAs were identified as *F. gigantica-* and *F*. *hepatica*-specific, respectively. For *F. gigantica*, one of these miRNAs matched with miR-4006b (cin-miR-4006b) of *Ciona intestinalis*, while for *F*. *hepatica*, one of these miRNAs matched with miR-1957 (mmu-miR-1957) of *Mus musculus*. The remaining miRNA candidates have not had match with known miRNAs, and were therefore novel miRNAs.

**Figure 1:**
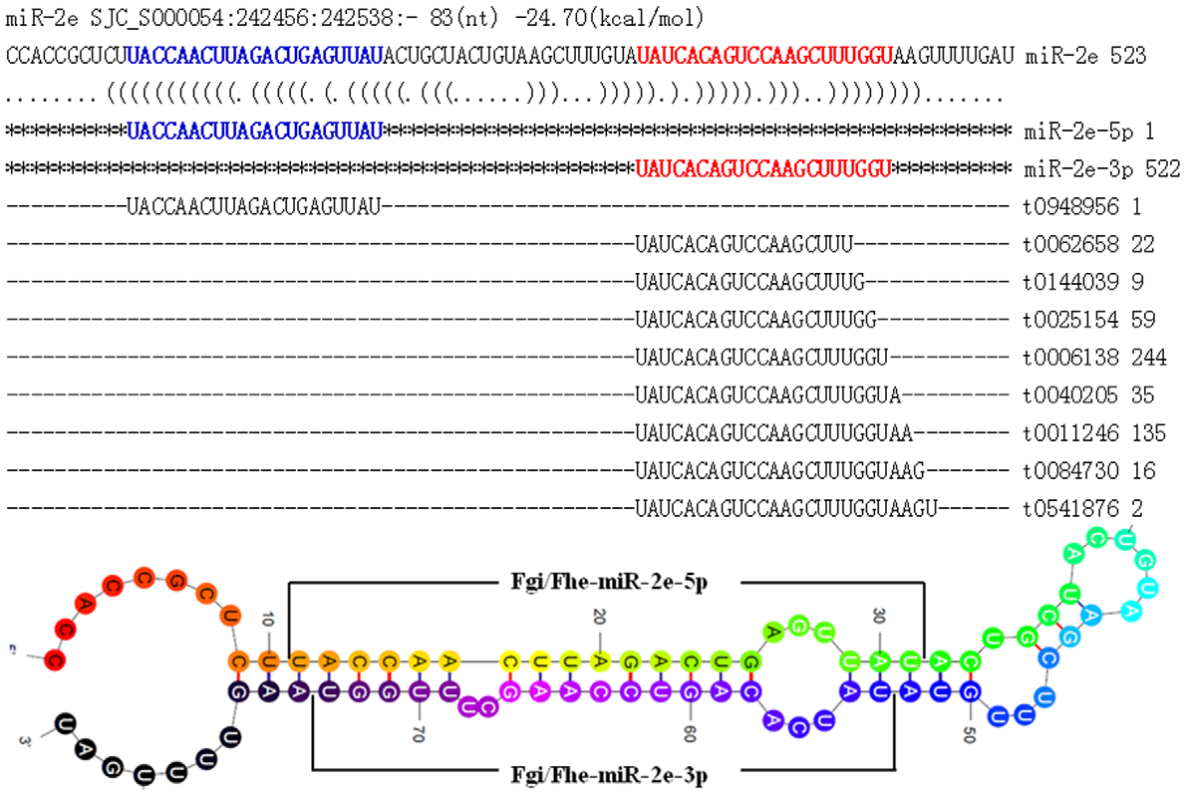
Precusors, homologous and secondary structure of miR-2e shared by *Fasciola hepatica* and *F. gigantica*. Upper: BLAST alignment of miR-2e. Red and blue show the miR-2e-3p and miR-2e-5p. Lower: stem-loop structure of the miR-2e precursor with the miRNA region indicated, colouring from red to black shows the 5′ to 3′ sequences.

A distinguishing characteristic of the miRNAs was that most precusors had only one mature miRNA originating from the 5p or the 3p arm, respectively, except miR-2e which had mature miRNAs on both arms of its precursor. However, although the miR-2e had a star sequence, its star sequence had only one copy (Figure 1).

### Target and Function Prediction

A total of 11465 mRNA and EST sequences from combined databases representing *F. hepatica* and *F. gigantica*, were used for target prediction of all miRNA candidates. It was found that all of the miRNAs matched with more than one target, ranging from 2 to 398, with an average of 51 targets (Table S1). For *F. gigantica*, some miRNAs had significantly high target numbers. For example, 3 novel miRNAs had target numbers of 114, 236, and 398. The accession numbers of targets are shown in Table S1.

**Figure 2:**
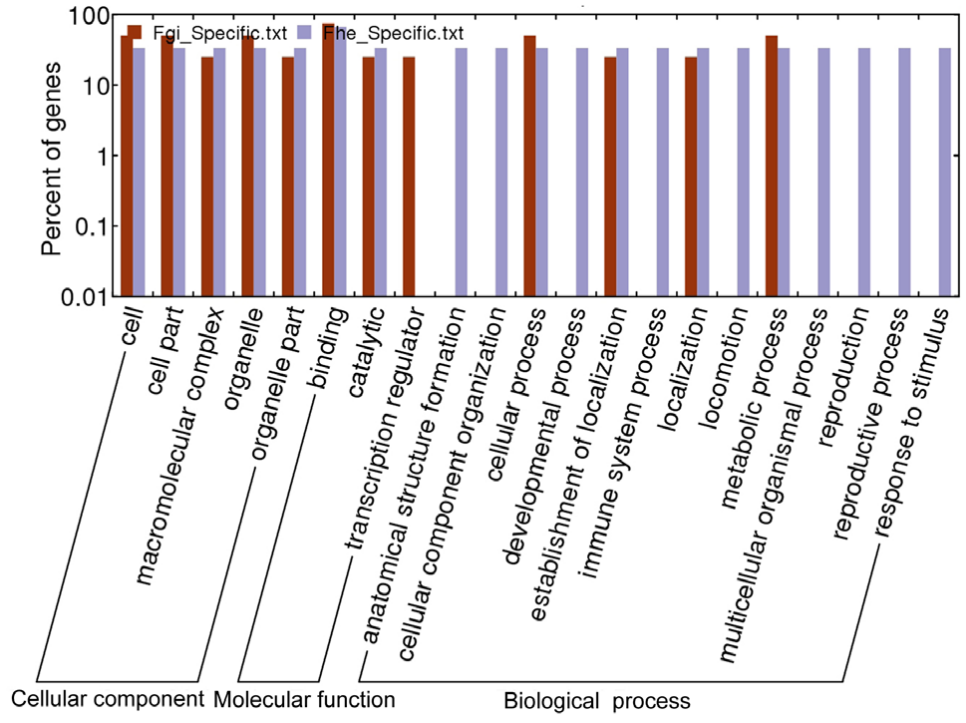
Comparison of functions of the best matched predicted targets.

To identify miRNA differences between the two parasites, the best matched targets of the species-specific miRNAs of each parasite were selected for functional prediction and comparative analysis. All the targets in *F. hepatica* and *F. gigantica* were similar in catalytic and binding functions, but “transcription regulator” function was only found in the targets of *F. gigantica*. There were some significant differences in the biological processes of the identified targets: Metabolic process were similar between the two parasites, but some other processes, including immune system processing, locomotion, reproduction and response to stimulus, were only found in the targets of *F*. *hepatica* (Figure 2).

### miRNA Quantification

**Figure 3:**
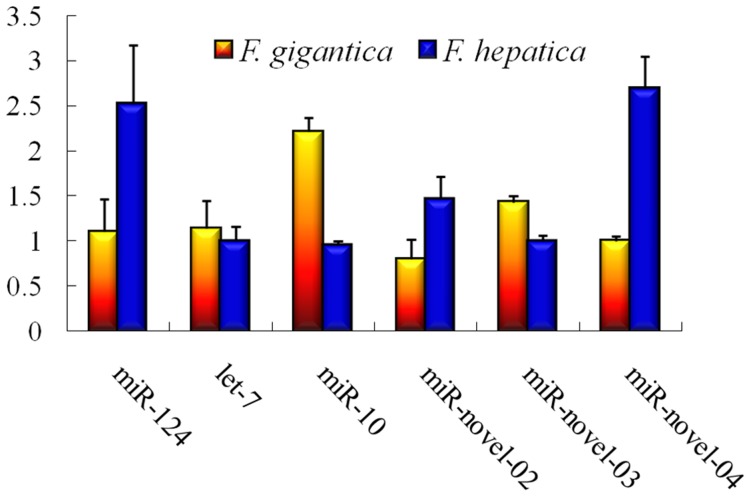
Relative expression levels of miRNAs in *Fasciola hepatica* and *F. gigantica*. The relative expression levels of miRNAs were calculated with cel-lin-4 as an internal reference, bars with different colours show the mean expression levels of each miRNA in *F. hepatica* and *F. gigantica*. Error bars show standard deviation (SD) for triplicate independent reactions.

The 6 novel miRNAs shared by the two parasites were selected for qRT-PCR analysis (Figure 3). The relative expression levels (REL) of three miRNAs were higher in *F. hepatica* than in *F. gigantica*: REL of miR-124 was 2.52±0.65 in *F. hepatica*, which was 2.29 times higher than that in *F. gigantica* (1.10±0.36); REL of miR-novel-04 was 2.69±0.36 in *F. hepatica*, which was 2.69 times higher than that in *F. gigantica* (1.00±0.05); REL of miR-novel-02 was 1.83 times higher in *F. hepatica* (1.46±0.25) than in *F. gigantica* (0.08±0.21).

REL of the other 3 miRNAs was found to be lower in *F. hepatica* than that in *F. gigantica*. The REL of miR-10 was 2.33 fold higher in *F. gigantica* (2.21±0.16) than that in *F. hepatica* (0.95±0.05); and the let-7 was 1.14 fold higher *F. gigantica* (1.14±0.30) than that in *F. hepatica* (1.00±0.16). For miR-novel-03, a 1.43 times higher expression level was found in *F. gigantica* (1.43±0.07) than in *F. hepatica* (1.00±0.06).

## Discussion

In the present study, we obtained 16.41 and 10.36 million high quality reads greater than 18 nt from the total RNA of *F. hepatica* and *F. gigantica*, respectively. Among these reads, 8.7 and 3.5 million reads were *F. hepatica-* and *F. gigantica-*specific, which represented 2.9 and 0.48 million unique reads, indicating 6.04 times higher numbers of unique reads from *F. hepatica* than from *F. gigantica*. However, the numbers of miRNA candidates were similar between the two species, with 19 and 16 miRNA candidates identified in *F*. *gigantica* and *F*. *hepatica*, respectively. This phenomenon indicated a higher level of redundancy in the expression of small RNAs in *F. hepatica* than in *F. gigantica*, and further indicated a potential different mechanisms of gene regulation between the two parasites.

Most of the miRNAs were shared by *F. gigantica* (57.89%, 11/19) and *F. hepatica* (68.75%, 11/16) and most of the shared miRNAs (72.73%, 8/11) were fully conserved with miRNA sequences from *S*. *japonicum* as opposed to miRNAs from other species deposited in the miRBase database. When miRNAs of another trematode species *Schistosoma mansoni* [20], were included for analyses, an additional miRNA (sma-miR-125a) was identified as being commonly expressed in *F. gigantica*, *F. hepatica* and *S. mansoni*. As *S*. *japonicum*, *S. mansoni*, *F. gigantica* and *F. hepatica* are all trematodes, they are likely to have some similar metabolic processes and therefore share similar miRNAs. In terms of species-specific miRNAs, only one miRNA from each species could be identified which possessed a known homologue in other species. This phenomenon might indicate species-specific characteristics of the miRNAs of the two parasites compared with other parasites. It has been reported that an “intermediate genotype” of *Fasciola* spp. might be a hybrid of the two species [5], [27]. We speculate that the intermediate-*Fasciola* may contain “species-specific” miRNAs from *F. gigantica* and *F. hepatica*, because significant regulatory mechanisms of each species would be genetically inherited by the hybrids. Furthermore, there may also be some intermediate-*Fasciola* specific miRNAs emerging, because of the key regulatory, and rapidly evolving characteristics of miRNAs, as we have described from other parasites including *Clonorchis sinensis* and *Orientobilharzia turkestanicum* [19], [28].

Target prediction analysis showed that some of the miRNAs had substantially higher target numbers than others in *F. gigantica* with 114, 236, and 398, whereas the three highest target number of miRNAs of *F*. *hepatica* were 64 (Fhe-miR-novel-10), 39 (Fhe-miR-novel-07) and 23 (Fhe-mmu-miR-1957). One reason for this phenomenon might be that there were more mRNA and EST sequences available for *F. gigantica* (8410) than for *F. hepatica* (3055). To attempt to overcome this limitation, the mRNA and EST sequences of *F. gigantica* and *F. hepatica* were combined before target prediction. Moreover, the highest target number of *F. gigantica* was 6.22 times higher than that of *F. hepatica*, while *F. gigantica* mRNA and EST sequences represented only 2.75 times higher numbers in the combined interrogated dataset. Overall, target analysis indicated that the miRNA Fgi-miR-novel-03 had more targets than any of the others, which might indicate higher importance of the miRNA for the parasite than other miRNAs. This phenomenon can be further verified by the genome copy number of Fgi-miR-novel-03: it occurs in six locations on the reference genome, while the others are only represented one or twice. Increased copy number within a genome is an effective approach to improve the expression of a regulator [29], [30]. This result therefore indicated an obvious regulating difference of miRNAs of the two parasites. Furthermore, the metabolic differences between the two parasites were verified by the analysis of the functions of predicted targets: some targets and functions were found to be *F. gigantica-*specific, while some others were *F. hepatica-*specific.

In conclusion, the present study represents the first global comparative characterization of miRNA expression profiles of *F. gigantica* and *F. hepatica*, and has provided a novel, unique resource to facilitate fundamental and applied molecular investigations of the two key representatives of liver flukes, which in turn has implications for the better control of human and animal fasciolosis.

## Supporting Information

Table S1 **Target number and complementary structure of predicted miRNAs.**
(DOCX)
