# Supplementary material for: Comparative Characterization of MicroRNAs from the Liver Flukes Fasciola gigantica and F. hepatica
Source: PLoS One. 2012 Dec 31;7(12):e53387. doi: 10.1371/journal.pone.0053387 (PMC3534066; doi:10.1371/journal.pone.0053387)
Supplement: Table S1 — Target number and complementary structure of predicted miRNAs. (DOCX) [file pone.0053387.s001.docx]

| **Name** | **Length** | **Best Match** | **Mfe**  **(kcal/mol)** | **Total**  **target** | **Complimentary Structure** |
| --- | --- | --- | --- | --- | --- |
| **Shared miRNA** |  |  |  |  |  |
| sja-bantam | 22 | gi\|328499975 | -33.9 | 8 | target 5' **C C G C** 3' |
|  |  |  |  |  | **GCC GCUUUGGUCG GAUCUC** |
|  |  |  |  |  | **UGG CGAAAUUAGC CUAGAG** |
|  |  |  |  |  | miRNA 3'  **U G U** 5' |
|  |  |  |  |  |  |
| sja-let-7 | 20 | gi\|328503371 | -32.3 | 37 | target 5'  **G G A** 3' |
|  |  |  |  |  | **A GCAGCGGACUACUUCC** |
|  |  |  |  |  | **U UGUUGCUUGAUGGAGG** |
|  |  |  |  |  | miRNA 3' **GG G**  5' |
|  |  |  |  |  |  |
| sja-miR-10-5p | 21 | gi\|53552843 | -33.4 | 15 | target 5'  **C U G** 3' |
|  |  |  |  |  | **GAUUCGGG UCUACGGGG** |
|  |  |  |  |  | **UUGAGCCC AGAUGUCCC** |
|  |  |  |  |  | miRNA 3' **GU AA** 5' |
|  |  |  |  |  |  |
| sja-miR-124-3p | 21 | gi\|328499580 | -32.9 | 38 | target 5' **U U A** 3' |
|  |  |  |  |  | **CAUUCACC CGUGCCUUA** |
|  |  |  |  |  | **GUAAGUGG GCACGGAAU** |
|  |  |  |  |  | miRNA 3' **ACU C**  5' |
|  |  |  |  |  |  |
| sja-miR-2b-3p | 23 | gi\|328500187 | -37.1 | 40 | target 5' **A A** 3' |
|  |  |  |  |  | **GUGUCCUGAGCAGGGC** |
|  |  |  |  |  | **CACAGGGUUCGUCCCG** |
|  |  |  |  |  | miRNA 3'  **ACACUAU** 5' |
|  |  |  |  |  |  |
| sja-miR-2e-3p | 20 | gi\|294845651 | -28 | 16 | target 5'  **C C U** 3' |
|  |  |  |  |  | **AAGCU GGACUGUGAU** |
|  |  |  |  |  | **UUCGA CCUGACACUA** |
|  |  |  |  |  | miRNA 3' **GGU A U** 5' |
|  |  |  |  |  |  |
| sja-miR-71-5p | 22 | gi\|328506372 | -31.1 | 15 | target 5' **G G**  3' |
|  |  |  |  |  | **GUCUCACUGCCAUUGU** |
|  |  |  |  |  | **UAGAGUGAUGGUAGCA** |
|  |  |  |  |  | miRNA 3'  **GAAAGU** 5' |
|  |  |  |  |  |  |
| sja-miR-71b-5p | 23 | gi\|328502497 | -30 | 2 | target 5' **A G** 3' |
|  |  |  |  |  | **CG UUCGCU CUCAAGUUUUUCA** |
|  |  |  |  |  | **GC GAGUGA GAGUUCAGAAAGU** |
|  |  |  |  |  | miRNA 3'  **A U**  5' |
|  |  |  |  |  |  |
| miR-nov-02 | 20 | gi\|328503701 | -28.3 | 3 | target 5' **A U U**  3' |
|  |  |  |  |  | **CGCUGUACA GCUGUUUU** |
|  |  |  |  |  | **GUGACAUGU CGACAAAG** |
|  |  |  |  |  | miRNA 3' **C**   **UA** 5' |
|  |  |  |  |  |  |
| miR-nov-03-12 | 23 | gi\|328502328 | -42.2 | 11 | target 5' **U G U** 3' |
|  |  |  |  |  | **GAAACAGCUGUGCAGUGC UCCA** |
|  |  |  |  |  | **UUUUGUCGACAUGUCACG AGGU** |
|  |  |  |  |  | miRNA 3'  **A**  5' |
|  |  |  |  |  |  |
| miR-nov-047816-45611 | 23 | gi\|53552839 | -35.4 | 67 | target 5' **G G U C C U** 3' |
|  |  |  |  |  | **UCUGACC ACUG GU GUGGG GGC** |
|  |  |  |  |  | **AGACUGG UGAC CG UACCU CCG** |
|  |  |  |  |  | miRNA 3'  **U A**  5' |
| ***Fasciola gigantica* Specific** |  |  |  |  |  |
|  |  |  |  |  |  |
| DN-cin-miR-4006b-5p | 20 | gi\|294844317 | -28.6 | 2 | target 5' **G G G U** 3' |
|  |  |  |  |  | **CCUUG CUACAUUGU UCCG** |
|  |  |  |  |  | **GGAAU GAUGUAACA AGGU** |
|  |  |  |  |  | miRNA 3' **G G**  5' |
|  |  |  |  |  |  |
| DN-miRNA-novel-01 | 20 | gi\|328503083 | -32.3 | 41 | target 5' **U U** 3' |
|  |  |  |  |  | **GUCCAUCUGUCCGUCUGU** |
|  |  |  |  |  | **UAGGUAGAUAGGUAGGUA** |
|  |  |  |  |  | miRNA 3' **GG**  5' |
|  |  |  |  |  |  |
| DN-miRNA-novel-03 | 21 | gi\|328505430 | -41.8 | 398 | target 5'  **A A** 3' |
|  |  |  |  |  | **UAUUCGGCCACCCCGUC** |
|  |  |  |  |  | **GUGAGCCGGUGGGGCAG** |
|  |  |  |  |  | miRNA 3' **AUUG**  5' |
|  |  |  |  |  |  |
| DN-miRNA-novel-05 | 20 | gi\|328499880 | -34.1 | 13 | target 5' **C C G** 3' |
|  |  |  |  |  | **CCGAGGUGG G GAGGUGGU** |
|  |  |  |  |  | **GGCUCCAUC C CUUCACCA** |
|  |  |  |  |  | miRNA 3'  **A G** 5' |
|  |  |  |  |  |  |
| DN-miRNA-novel-06 | 20 | gi\|328500592 | -32.9 | 114 | target 5' **U C C C G** 3' |
|  |  |  |  |  | **UCGA CCAC UCACC ACCUC** |
|  |  |  |  |  | **AGCU GGUG AGUGG UGGAG** |
|  |  |  |  |  | miRNA 3'  **A A**  5' |
|  |  |  |  |  |  |
| DN-miRNA-novel-09 | 20 | gi\|294844317 | -28.6 | 2 | target 5' **G G G U** 3' |
|  |  |  |  |  | **CCUUG CUACAUUGU UCCG** |
|  |  |  |  |  | **GGAAU GAUGUAACA AGGU** |
|  |  |  |  |  | miRNA 3' **G G**  5' |
|  |  |  |  |  |  |
| DN-miRNA-novel-10 | 24 | gi\|53551804 | -43.2 | 31 | target 5' **U U** 3' |
|  |  |  |  |  | **GAUGAUGAUGAUGAUGAUGAUGG** |
|  |  |  |  |  | **CUACUACUACUACUACUACUACC** |
|  |  |  |  |  | miRNA 3' U 5' |
|  |  |  |  |  |  |
| DN-miRNA-novel-15 | 22 | gi\|328505931 | -48.3 | 236 | target 5' **C A 3**' |
|  |  |  |  |  | **UCUCUCUCUCUCUCUCUCUCUC** |
|  |  |  |  |  | **AGAGAGGGAGAGAGAGAGAGAG** |
|  |  |  |  |  | miRNA 3' 5' |
|  |  |  |  |  |  |
| ***Fasciola hepatica* Specific** |  |  |  |  |  |
| GP-mmu-miR-1957 | 21 | gi\|328500845 | -30.7 | 23 | target 5' **U U G A** 3' |
|  |  |  |  |  | **UCUG UGCU UAUCGACUG** |
|  |  |  |  |  | **AGAC ACGA AUGGCUGAC** |
|  |  |  |  |  | miRNA 3' **UC U G**  5' |
|  |  |  |  |  |  |
| GP-miRNA-novel-01 | 21 | gi\|328498066 | -28.6 | 4 | target 5' **C A G A**  3' |
|  |  |  |  |  | **GAAGCAC GUA AGUUGU** |
|  |  |  |  |  | **CUUCGUG CAU UCGACA** |
|  |  |  |  |  | miRNA 3' **U A G AA** 5' |
|  |  |  |  |  |  |
| GP-miRNA-novel-07 | 22 | gi\|328501456 | -36.2 | 39 | target 5'  **C U** 3' |
|  |  |  |  |  | **AUCGUCAUCAUCAUCAUCGU** |
|  |  |  |  |  | **UAGUAGUAGUAGUAGUAGCA** |
|  |  |  |  |  | miRNA 3' **UU**  5' |
|  |  |  |  |  |  |
| GP-miRNA-novel-08-9 | 21 | gi\|328499228 | -32.3 | 7 | target 5' **U U** 3' |
|  |  |  |  |  | **GGCAGUGGAUCCAUCAUC** |
|  |  |  |  |  | **UUGUCAUUUAGGUAGUAG** |
|  |  |  |  |  | miRNA 3' **UG U** 5' |
|  |  |  |  |  |  |
| GP-miRNA-novel-10 | 21 | gi\|328499044 | -33.4 | 64 | target 5' **U A U** 3' |
|  |  |  |  |  | **GAUCCUG AGCUGCUCCAUU** |
|  |  |  |  |  | **CUGGGGU UCGAUGAGGUAG** |
|  |  |  |  |  | miRNA 3' **U A**  5' |
